# Gender equality is crucial to the fight for better HIV treatment access and outcomes in the MENA region

**DOI:** 10.1002/jia2.25092

**Published:** 2018-03-05

**Authors:** Navid Madani

**Affiliations:** ^1^ Department of Cancer Immunology and Virology Dana‐Farber Cancer Institute Boston MA USA; ^2^ Department of Global Health and Social Medicine Department of Microbiology and Immunobiology Harvard Medical School Boston MA USA

**Keywords:** women, HIV, Middle East and North Africa, treatment, policy, education

In the past 37 years, HIV/AIDS researchers have profoundly advanced our understanding of the molecular biology, biochemistry and immunology of HIV thanks in large part to the urgency, engagement, and full mobilisation of different sectors of society. We have initiated long‐standing inter‐ and intra‐disciplinary collaborations allowing us to understand the biology and mechanisms of this virus, while expanding drug availability and patient advocacy.

HIV brought us out of our silos; in addition to being physicians and scientists, we became advocates, human rights activists and educators. Life‐saving treatment has been discovered, improved and disseminated around the world. With education, prevention and adherence to treatment, HIV infection became a chronic disease rather than an acute one, and for this reason, the epidemic has levelled off or is decreasing throughout the world today.

With a notable exception: the Middle East and North Africa (MENA).

The MENA region has an expanding HIV epidemic 1. In 2016, this region was one of only two worldwide in which AIDS‐related deaths rose by 19% or more 1. This is largely due to poor access to antiretroviral treatment (ART), with only 24% of individuals needing ART in the region having access to treatment, a figure well below the global level of 54% 1. Extreme HIV stigma, political conflicts and wars, socio‐economic factors, and lack of education are some of the reasons for the persistence of such barriers despite the expanding epidemic.

In this fight, women are particularly susceptible to the HIV infection. Not only are women more biologically prone to acquiring the infection 2, 3, but endemic interpersonal violence against women and girls, misogynistic traditions, lack of control over condom use and limited sex education reinforce unequal power dynamics between men and women.

While MENA women have made great strides towards education and health, the countries in the region, with one exception, still rank in the bottom 20% of the global health gender gap and continue to rank last on the overall gender gap index, behind South Asia 4. Yet what I see in my travels and in the educational workshops that I conduct in the region is that 90 to 95% of participants in these workshops are engaged and empowered women who are eager to learn, advance their careers and compete in the global forum.

Where is the disconnect? Why the low rankings in global statistics and increased susceptibility to diseases such as HIV/AIDS, despite my first‐hand experience of engaged and empowered women?

As a woman of Middle Eastern descent, I believe that the culture of inequality and disparity results in women self‐stigmatizing their positions and suffering from imposter syndrome—this too I have first‐hand experience with as I also dealt with demons of self‐doubt. But what is the solution to this self‐perpetuating cycle of social inequality that feeds women's self‐doubts and discourages them from breaking down social barriers?

Just as the urgency of dealing with HIV forced our global society to collectively deal with the epidemic, the still‐active epidemic in MENA requires us to look beyond our respective silos. In order to increase HIV treatment coverage among women, we need an effective and comprehensive healthcare system, which means including women in all sectors of this system. We need funding, education, and data‐driven leadership and decision‐making. And in order for all of the above to happen, we need education.

In MENA, community health workers form the backbone of health services; in many cases, these health workers are women. Why not start there? Why not increase education, self‐sufficiency and pay among these healthcare workers and providers? As a biochemist who is trying to develop a woman‐controlled microbicide against HIV transmission, I believe it seems crucial to ensure that women have the capacity to take control of their own healthcare—and not simply in terms of the ability to medicate themselves, but also in terms of running the very systems that will serve them. Such a focus would not only improve women's access to healthcare and life‐saving treatment, but also women's roles in society at large.

In 2002, when HIV transmission in women surpassed that of men, many in HIV communities and media wrote that HIV had “a woman's face” or had been “feminized” 5. I would argue that in MENA as well as worldwide, HIV has had a woman's face from the beginning of our fight against this devastating disease: the faces of mothers and sisters and daughters of the haemophiliac patients and injecting drug users who were the first to be diagnosed with HIV, and who stood by them and fought for better care and treatment. Faces like those of Professors Minoo Mohraz and Hakima Himmich, who were the mothers of response against the virus in their respective countries of Iran and Morocco and who fought for HIV care and treatment and better policies from the beginning of the epidemic, or those of a new generation of young female advocates in the region who fight for better reproductive health education and dialogue 6.

As we celebrate International Women's Day, I write this viewpoint in honour of these women, and the many others who came before and will follow as well as the women who are in the trenches now. I call on all scientists, physicians, advocates and educators to press for social equality, inform our societies as a whole, educate our young daughters AND sons to become better informed and tolerant world citizens. It is time for the world to press for inclusion and select informed religious and political leaders; and most importantly at this stage in our fight, to press for evidence‐based scientific discoveries and better treatment coverage.

In this fight, gender equality is crucial. We cannot make that progress unless all people in MENA, men and women alike, are equally involved.

## Competing interests

There are no competing interests.
